# Array of Hall Effect Sensors for Linear Positioning of a Magnet Independently of Its Strength Variation. A Case Study: Monitoring Milk Yield during Milking in Goats

**DOI:** 10.3390/s130608000

**Published:** 2013-06-21

**Authors:** Fernando-Juan García-Diego, Angel Sánchez-Quinche, Paloma Merello, Pedro Beltrán, Cristófol Peris

**Affiliations:** 1 Departamento de Física Aplicada (U.D. Industriales), Universitat Politècnica de València, Av. de los Naranjos s/n, 46022 Valencia, Spain; E-Mails: palomamerello@outlook.com (P.M.); pbeltran@fis.upv.es (P.B.); 2 Centro de Tecnologías Físicas, Unidad Asociada ICMM-CSIC/UPV, Universitat Politècnica de València, Av. de los Naranjos s/n. 46022 Valencia, Spain; 3 Instituto de Ciencia I Tecnología Animal, Universitat Politècnica de València, Av. de los Naranjos s/n, 46022 València, Spain; E-Mails: angeltigre1981@hotmail.com (A.S.-Q.); cperis@dca.upv.es (C.P.); 4 Instituto Valenciano de Conservación y Restauración de Bienes Culturales (IVC+R), Complejo Socio-Educativo de Penyeta Roja s/n, 12080 Castellón, Spain

**Keywords:** Hall effect sensors, milk yield, multiple regression, linear magnet position

## Abstract

In this study we propose an electronic system for linear positioning of a magnet independent of its modulus, which could vary because of aging, different fabrication process, *etc.* The system comprises a linear array of 24 Hall Effect sensors of proportional response. The data from all sensors are subject to a pretreatment (normalization) by row (position) making them independent on the temporary variation of its magnetic field strength. We analyze the particular case of the individual flow in milking of goats. The multiple regression analysis allowed us to calibrate the electronic system with a percentage of explanation R^2^ = 99.96%. In our case, the uncertainty in the linear position of the magnet is 0.51 mm that represents 0.019 L of goat milk. The test in farm compared the results obtained by direct reading of the volume with those obtained by the proposed electronic calibrated system, achieving a percentage of explanation of 99.05%.

## 1. Introduction

Currently, there is a great interest in automating the flow measurements and total milk production in milking of goats. With milk flow record it is possible to characterize the kinetic of milk emission, aspect of physiological interest [1] which traditionally has been manually performed [2]. Moreover, control of total milk yield per animal, fundamental variable in genetic improvement schemes [3], is performed by the official milk control agencies using volumetric meters, proportional meters or automated systems.

Automation of milk recording in dairy small ruminants may be a way of reducing costs and human errors [4,5] due to the large number of animals processed on the test-days. In goats, one of the most widely used meters is WB Mini-Test (Tru-Test), which operates by collecting a cumulative proportion (about 1/20) of the milk yield from an animal in a cylindrical container, with a capacity of approximately 300 mL, and the user performs a visual reading of a graduated scale to obtain an estimate of the total milk yield. In goats, this equipment allows to carry out measurements up to 5.5 L of milk yield per animal. There are currently several automatic milk meters to measure the flow and production of goats' milk, but we should point out that in many countries there are hardly used by official milk control agencies in goats' milk, either because they are fixed and cannot be transported routinely to farms (Afifree, MM25SG), or because of their high cost (Lactocorder). In practice few commercial goat farms have implemented automatic milk recording systems due to the high acquisition costs of the equipment [6].

Currently, there are two ways of determining magnetic fields vector distributions: (i) using a scanning machine [7,8]; (ii) using an array of sensors [9–12]. The first type of device offers a high special resolution, but their structure is complicated and they require a longer time than array type magnetic sensor for measuring magnetic fields vectors. There is another hybrid type [13] that is a scanning with an array of sensors to increase the resolution.

All cited works try to develop a 1, 2 or 3 dimension camera to determine the magnetic field vectors. In this work we try to develop an array of magnetic Hall Effect sensors to determine the position of the magnet. The aim of this work is to design a level meter, of low-cost, to be used in combination with the proportional meter WB Mini-Test, and thus automate the record of the flow and total milk production in milking of goats.

The proposed electronic system surmount the inherent difficulties in these measures: (i) the milk composition and so the physico-chemical properties of the milk vary between animals; (ii) within the milk meter the milk is subjected to vacuum (about 35–40 kPa) and foaming may occur; (iii) the meter is subjected to washing with acid and basic detergents and hot water (50–70 °C), as well as all components added into the milk flow.

In this study, with the intention of overcome the above described obstacles, we propose a milk volume meter where the entire electronic components are positioned outside the milk flow. This is achieved because the proposed volume meter is based on the detection of the variation of the magnetic field created by a magnet placed on a floating device and an array of linear Hall Effect sensors.

The magnets may age changing the strength of its magnetic field. This causes that the calibration of a Hall Effect sensor with linear response would not be lasting. This paper also proposes a basic statistical methodology to overcome this problem.

## 2. Materials and Methods

In this section, the developed measurement system, as well as the methodology followed for the magnetic field calibration, will be described.

### 2.1. Array of Sensors

**Figure 1.**
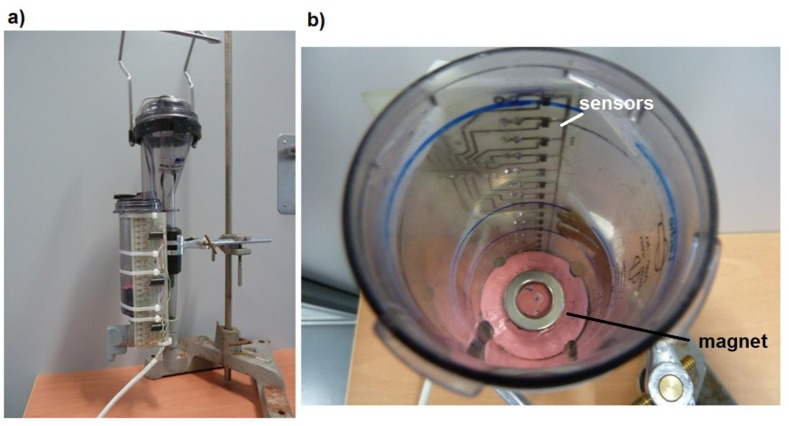
Photographs of the electronic system and its location in the proportional meter, **(a)** front view of the proportional meter whit the electronic circuit attached, **(b)** view from above of dismounted receiving container, showing part of the array of Hall Effect sensors and the magnet over the pink float.

The proposed system consists of an electronic board attached to the proportional meter WB Mini-Test, where Hall Effect sensors are welded vertically. Inside the proportional meter, a floating device containing a magnet is placed, so that the reading of the Hall Effect sensors will vary as the float raises its height, as a result of the volume increase (Figure 1).

The Hall Effect sensor chosen is A1301 [14], which is optimized to accurately provide a voltage output proportional to the flux of the applied magnetic field which cross the sensor. These devices have a quiescent output voltage (2.5 V in our case), which is the half of the applied one. With an output sensitivity of 2.5 mV/G, high precision in output levels are obtained by internal gain and offset trim adjustments made at end-of-line during the manufacturing process.

They are well-suited for industrial applications; it is immune to vibrations end extended temperature ranges (from −40 °C to 125 °C). These features make the A1301 ideal for use in linear position sensing systems [14]. The device package was a 3-pin SOT23W type for surface mount, this package has a better stability in temperature changes than other ones [14].

An array of 24 sensors (A1301), placed with a linear separation of 8 mm, was constructed. The total length of the array of sensors was of 184 mm. Because of constructional reasons, the upper sensor was placed close to the edge of proportional measurement device. A total of five sensors protruded below the lower edge of the container. The array was placed on the outside of the cylindrical milking measuring device, parallel to its axis, so that its internal washing with acid and basic detergents and hot water does not interfere in the array.

**Figure 2.**
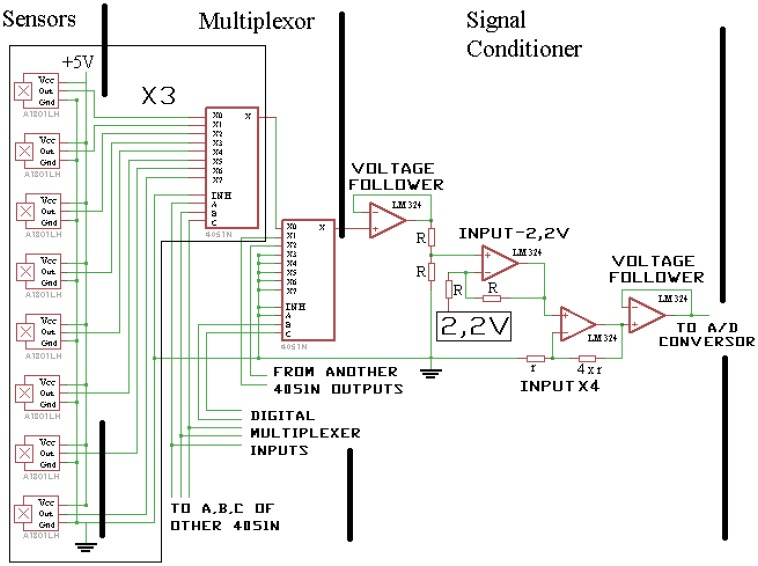
Electronic sketch of the array of sensors, multiplexing and conditioning of the signal.

As shown in Figure 2 the array was multiplexed with four 8-channel analog multiplexer (4051N) with the intention of obtaining a single signal, which was conditioned by four operational amplifiers contained in a LM324. The four operational amplifiers were connected in the following order: the first as a voltage follower, the second subtracts 2.2 V to the input signal, the third multiplies the second signal by five, and the last one follows the tension of the third amplifier.

The analog signal is read by a 10-bit ADC (Analogue to Digital Converter), from an ATMEGA168 (Atmel, San Jose, CA, USA) microcontroller. Five digital pins of the microcontroller were used to manage the multiplexors. The microcontroller was programmed using the Arduino [15] Integrated Development Environment (IDE). The microcontroller saved milking data in a USB memory with a frequency programmable by the user.

To create the magnetic field, a permanent magnet shaped washer, to lighten its weight, was selected. A NdFeB magnet [16], with 26.75/16 mm diameter (outside/inside, respectively), with 5 mm in height, with their north and south poles on the flat circular surfaces (axial magnetisation), 14 g in weight, and a nickel plated (Ni-Cu-Ni), is capable of withstanding temperatures of 80 °C. The magnet was attached to the upper base of a cylindrical floating device, in PVC, with 52 mm diameter and 50 mm high, and a weight of 70 g. The floating device has vertical grooves to make the milk flowing up and down. As the float fitted perfectly into proportional measuring device the floating device requires a minimal volume (50 mL) to start floating.

**Figure 3.**
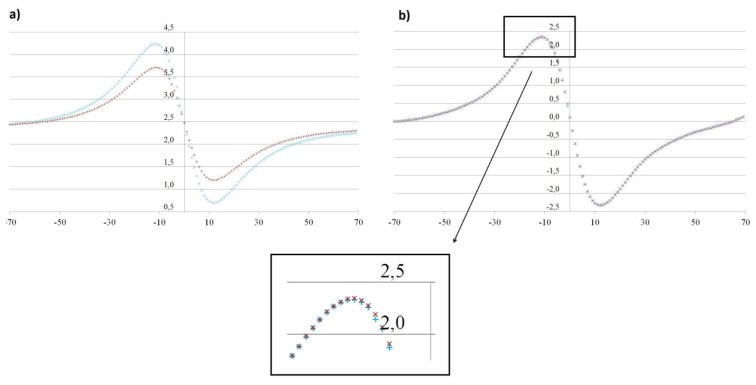
**(a)** Potential in volts (vertical axis) as a function of the position (mm) of the magnet with respect to a sensor in the center of the array (horizontal axis). Graphic in blue corresponds to the magnet without heat treatment, the red one corresponds to the magnet with heat treated. **(b)** Normalized value of the potential, for both magnet without heat treatment (blue) and treated (red). A value of zero on the horizontal axis indicates coincidence of positions (position of the sensor equal to the magnet position). We have made a zoom to see the difference between both points.

The magnetic field created by a permanent magnet is explained elsewhere [17,18]. The magnetic field produced by the magnet is not distorted by the measuring system because all was made of non-ferromagnetic materials (plastic, stainless steel, copper and tin). In our case, we are interested only in the radial component of the magnetic field, which will vary the flow of the magnetic field on our sensors. A result greater than 2.5 V is obtained when the magnet surpasses the sensor, and score less than 2.5 V is obtained when the magnet is below the sensor, as shown in Figure 3(a). In a commercial device, the magnet could be placed into the PVC floating device or painted with a suitable paint to prevent rusting.

### 2.2. Experimental Design for Calibration

The scale of the original proportional meter represents a total volume of 5.5 L of milking. Given the height of the floating device, which contains the magnet, the final proportional meter is capable of measuring 4.07 L, more than enough for the Murciano-Grenadine goats used in this experiment because they do not exceed 2 L of total milking [6]. For the calibration procedure, these 4.07 L were divided into 110 parts, each one of 1 mm. The float moves jointly with a plastic metric screw of 6 mm of diameter, which each screw turn lifts the float 1 mm (0.037 L). Goat's milk was manually added to the cuvette as the float height was increased. Each experiment consists of 110 measures for each of the 24 sensors.

There were two batches of different experiments, each one consisting of eight individual experiments. Since the meter is subjected to proportional washed with hot water (50–70 °C), the first batch was carried out to check the effects on the heat treatment on the magnet and how data pretreatment (Section 2.3) overcomes this problem. Three of these eight initial experiments were made after subjecting the magnet to a heat treatment at 80 °C for 5 minutes.

The second batch was made for final calibration of the system and checking the effects of different fat content in milk. Of the eight experiments, which were used to perform the final calibration system, five experiments were performed with whole cream milk and three with the same milk after a skimming process to check, through a regression analysis, if the fat content of the milk appreciably alters the magnetic field strength detected by the sensors. Milk fat composition was determined by mid-range infrared spectroscopy using a MilkoScan FT120 (Foss, Hillerød, Denmark).

Note that, after verifying that the normalization eliminates the effects of heat treatment on the magnet (Section 3.1), the second batch of experiments, performed with milk with different fat content, were all made with the magnet without heat treatment.

Each batch was composed of a total of eight experiments, so that in this study there are two data matrices of 880 (8 × 110) rows and 25 columns (24 sensors and variable volume) each one. All analysis and subsequent sections work with the calibration of the eight experiments of the second batch.

### 2.3. Data and Pretreatment

The data were subjected to a pretreatment with the intention of obtaining the best possible results in calibration and to extend this approach to all situations in which an event, inherent or not to the monitoring system, affects the response of all the sensors at the same time without altering the shape of the magnetic field.

The pretreatment consisted of normalizing the data by rows, obtaining from a random variable of unknown distribution a new random variable with normal distribution N(0,1), a treatment used in basic statistics [19]:

$$S\prime_{i}\left(t\right)=\frac{S_{i}\left(t\right)-\mu \left(t\right)}{\sigma \left(t\right)}$$

where *S* ′ _*i*_(*t*) is the normalized value for that sensor at time *t*, *S_i_*(*t*) is the sensor signal obtained from the sensor at the instant *t*, and the other two parameters are the mean value of *P* recorded by all sensors in a given instant of time *t*(μ(*t*)) and standard deviation of all sensors at time *t*.

The data are centered so that the analysis is focused on the variability around the average. Data are scaled to homogenize the importance of the variables in the model. The evolution *vs.* time of μ(*t*) is of relevant interest for the identification of different kinetics of milk emission. This common pattern of μ(*t*) is followed by all sensors, and the goal in this case is to diagnose the slight variations of each sensor respect this average performance. Thus, if data variability caused by time, equally affecting the magnitude of the magnetic field for all volume values but not its shape and direction, is eliminated from the matrix, the subsequent analysis will reflect more clearly the relationships among sensors. For example, changes in magnet strength, temperature, *etc*. such as the magnet aging which affects the modulus (strength) of the magnetic field, but not its shape [17,18]. Furthermore, using centered variables can help reduce some types of multicollinearity [20]. The idea of applying this pretreatment comes from the literature on the multivariate statistical control of batch chemical processes [21-25]. For this purpose, we centered (*i.e.*, subtracting μ(*t*)from the elements of row *t*) and scaled the data to unit variance by rows (dividing each value by the standard deviation of the row), so that all centered rows have null average and standard deviation equal to 1.

### 2.4. Regression Analyses

The sensors return a 10-bit value (*P*) between 0 and 5 Volts (*i.e.*, 5 V is divided in 1,024 parts), however for the calibration and future practical use of the system, it is interesting to propose a model that allows to infer the value of total milk yield (*V*, in L) in time t from the returned value (*P*) of each sensor in the moment of time t.

Therefore, a multiple regression analysis (GLM, Generalized linear model) is performed, with dependent variable *V_f_*(*t*) manually measured every 0.037 L (1 mm in the float height) through the aforementioned procedure. The independent variables in the regression are the values of P for each sensor (*P_i_*) normalized as explained in Section 2.3.

A sample of 880 values was used to calibrate the system. For each calibration, the output volume (*V_f_*(*t*)) was measured as a linear function of Hall Effect sensors response (*P_i_*(*t*)), following Equation (1), where *π* represents the error. In the case of thermal treatment a variable dummy *d*, taking the value 1 when the magnet has a heat treatment and the value 0 otherwise. In the case of variable fat content the variable dummy *d*, takes the value 1 when the goat milk is skimmed and the value 0 for the case where it is whole cream, was also included:

(1) $${\text{V}}_{\text{f}}(\text{t})=\alpha +\gamma \;\text{d}+\beta_{\text{i}}\;{\text{P}}_{\text{i}}(\text{t})+\Omega_{\text{i}}\;\text{d}\;{\text{P}}_{\text{i}}(\text{t})+\pi ,\;\text{I}=1,\ldots ,\;24.$$

A least-squares algorithm is used to obtain the coefficients of the linear regression (*α*, β_i_, γ and Ω_i_) for the reference volume (*V_f_*(*t*)) measured by the procedure described in Section 2.2. This analysis was performed using the software Statgraphics 5.1 [26].

Note that some pairs of *P_i_* are significantly correlated. Multicollinearity (correlated predictor variables) is bad in regression because it reduces the precision of the parameter estimates. While it affects the estimation of the individual parameters (each sensor in this case), multicollinearity does not affect inferences regarding the full model [20]. In the present study, it is of interest an accurate prediction of *V*, despite the exact contribution of each sensor to the prediction, so the multicollinearity will not cause any inference problem.

### 2.5. Experimental Design in Farm

Once the electronic system was calibrated in the laboratory, its operation was validated under farm conditions. For this purpose, it was installed in the line of the milking machine in the experimental farm of the Universitat Politècnica de València. The (2×12) milking parlour used had six clusters (Almatic™ cluster G50, Delaval Agri, Tumba, Sweden) and a milk pipeline at 1.0 m above the platform (midlevel). Milking parameters were rate of 90 pulsations per minute, vacuum level of 40 kPa, and 60% pulsation ratio. Five goats of Murciano-Grenadine race were milked, with a milk production ranging between 1.2 and 4 L. The milk flow was recorded according to the method in [2], so that the volume milked was manually recorded (according to the scale of the proportional meter) every 5 s, while, at the same time, the milk production was recorded by the electronic system every second. The verticality of the apparatus was ensured by holding a weight of 1 kg of the WB Mini-Test (Tru-Test).

In order to avoid the problem of the minimum volume to which the floating device starts to float, which depends on its shape and not on the electronic system, the measuring of milk yield is started with an initial volume of 50 mL in the proportional measurement device. These 50 mL have been subtracted from all the results.

## 3. Results and Discussion

### 3.1. Calibration with Thermal Treatment

Initially a multiple regression analysis was performed with the data of the first batch of experiments, in which it was found that the applied pretreatment was able to neutralize the effect that a heat treatment of the magnet has on the magnetic field, resulting not a significant variable (dummy variable which takes the value 1 if the magnet has been heat treated and 0 otherwise, p-value>0.05).

Figure 3(a) shows the reduction in the sensor response after subjecting the magnet to a reversible heat treatment (decreased potency). Figure 3(a) also allows observing the excellent repeatability of the sensors, taking into account that each one of the paths is the superposition of 5 and 3 experiments for the case with thermal treatment of the magnet and without it, respectively.

This made the magnet lose intensity as shown in Figure 3(a). It was found with the graphs (Figure 3(b)) and an exploratory regression analysis that the normalized data showed no influence of thermal treatment.

### 3.2. Calibration with Cariable Fat Content

In later analyses have been performed on the second batch of experiments. Then, the multiple regression analysis has allowed obtaining a system calibration.

Milk fat content was 5.05% whole cream milk and 0.55% after skimmed process, measured by mid-range infrared spectroscopy. The total milking volume in each time *t* can be predicted according to Equation (1), with the parameter values. Sensors 1 and 5 were not significant with a p-value > 0.05. The dummy variable modeling the influence of the fat content was not significant (p-value > 0.05). The fact that sensor 1 is not significant evidences that there is a maximum distance from which the sensors cannot detect differences in magnetic field caused by the movement of the magnet and sensor 5 because it could have a lineal dependence whith other sensors of the array. The model was significant (p-value < 0.01), with an R^2^ de 99.962%, a mean absolute error of 0.019 L (average of the error) and a standard error of estimate of 0.024 L (standard deviation of the error).

A regression analysis to the data without amplification was also performed. For the signal without amplifier the mean absolute error was 0.023 L and the standard error of estimate was 0.029 L, so that amplify the signal has improved the model by reducing the error by approximately 18%.

**Figure 4.**
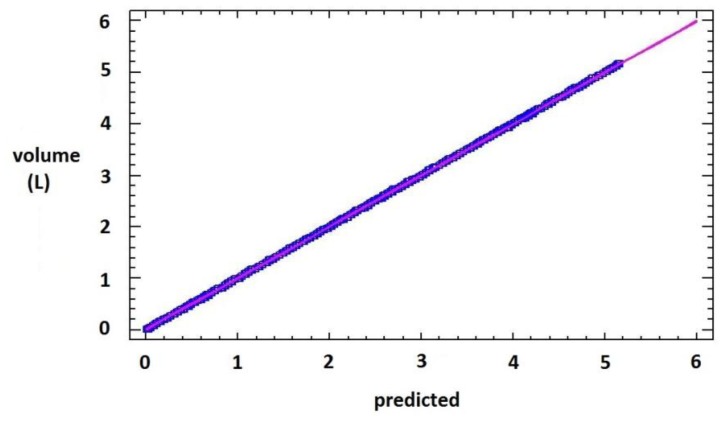
Output volume in L *vs.* predicted volume by the calibration curve (L).

A second linear regression analysis was performed, with dependent variable the manually measured volume (*V_f_*(*t*)) [2] and the predicted volume (*V_p_*(*t*)) by the calibration model (Table 1) as independent variable. The model fitting is shown in Figure 4, which is a significant model (P-value < 0.01), with intercept near cero and a slope near 1 in Equation (2), a percentage of explanation (R^2^) of 99.962%, and a mean absolute error of 0.054 L:

(2) $$V_{f}(t)=-3.95\times 10^{-8}+1\times V_{p}(t).$$

### 3.3. Verification and Validation in Farm

Once the calibration model is obtained for the electronic system considering the different sensors responses (Table 1), in order to ascertain the validity of the calibration a linear regression analysis between the manually measured volume in a practical experiment in the farm (*Vf*(*real*)) and the volume estimated by the proposed system (*Vf*(*e*)) is performed. The model is described by Equation (3), with intercept zero, slope 0.9931 ≈ 1 (Equation (3)), and an explanation percentage (R^2^) of 99.05%:

(3) $$\text{E}({\text{V}}_{\text{f}(\text{real})})=0.9931\;{\text{V}}_{\text{f}(\text{e}).}$$

The farm test has revealed the practical use of this system under real ambient conditions and milking kinetics. The calibration model (Table 1) allowed predicting a 99.05% of the variability of the data. A confidence interval of ±0.05 L was chosen because this is the half of the scale marks in the WB Mini-Test and it could be assumed as the operator error. Furthermore, the instrument error must be added but we could not found it in the builder data-sheet and is supposed to be much less.

**Figure 5.**
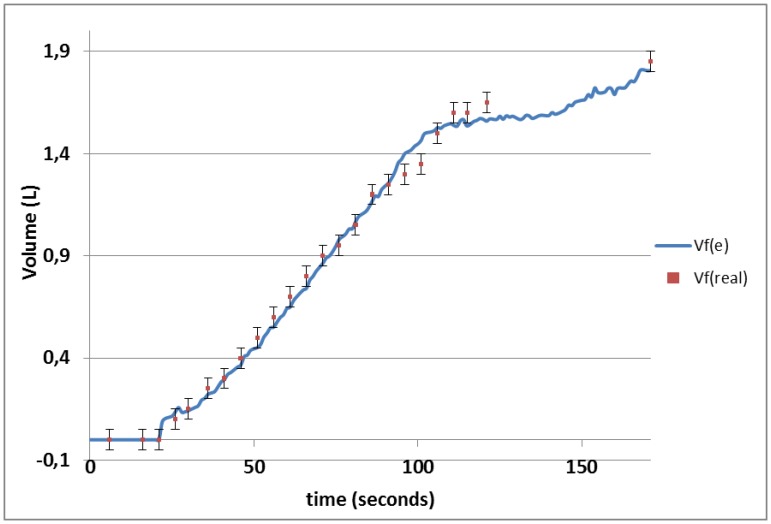
Manually measured volume (*Vf*(*real*)) and volume estimated by the electronic system and calibration model (*Vf*(*e*)) *vs.* time. The confidence intervals have been plotted for *Vf*(*real*) considering a manual measurement error of ±0.05 L.

Notice that the value predicted by the electronic system is contained within the confidence interval of the manual measurement in the 87.5% (21/24) of the cases (Figure 5). The electronic system could be improved by determining the number of sensors and optimal separation between them to get the best cost-adjustment percentage (R^2^) possible. These improvements will be studied in a future work.

## 4. Conclusions

We have designed an electronic system using Hall Effect sensors. The electronic system is low-cost and has a high portability and, thanks to a pretreatment performed by row on the data, it circumvents problems as changes in the magnetic field strength generated by heat treatment on the magnet (as might result after washing with hot water the proportional measurement device) or other cause of magnet aging.

The calibration of the system has achieved very successful results, with a percentage adjustment of 99.962% and a mean absolute error of 0.019 L. The fat content of the milk was not a significant factor in the response of the sensors. By means of a practical test in farm, the correct operation of the system under uncontrolled conditions and in direct use with animals has been proven. The system has achieved a prediction adjusted in a 99.05% to the volume measured manually from the proportional volumetric meter.

## Figures and Tables

**Table 1.** Parameter and its estimation (L/10-bit value (*P*)) of the significant variables of the multiple linear regression for all P-values that obtained a significance level lower than 0.01.

**Parameter**	**Estimation**	**Statistic T**	**P-Value**	**Parameter**	**Estimation**	**Statistic T**	**P-Value**
α	3.40876	55.8703	0.0000	β14	4.09285	14.8823	0.0000
β2	2.56316	4.55189	0.0059	β15	3.97483	14.4389	0.0000
β3	2.90016	5.04019	0.0001	β16	3.93641	14.31	0.0000
β4	3.44822	6.25207	0.0068	β17	3.84902	14.0004	0.0000
β6	5.57883	16.8217	0.0000	β18	3.85293	14.0064	0.0000
β7	3.03645	5.48769	0.0000	β19	3.80196	13.8264	0.0000
β8	7.39145	13.7871	0.0000	β20	3.73793	13.6	0.0000
β9	3.83972	9.00275	0.0000	β21	3.75852	13.6837	0.0000
β10	5.131	18.8555	0.0000	β22	3.64796	13.2721	0.0000
β11	3.94889	13.2547	0.0000	β23	3.75163	13.6819	0.0000
β12	4.28748	15.5634	0.0000	β24	3.65412	13.3037	0.0000
β13	4.16296	15.0817	0.0000				
